# Syphilis Infection Triggering Autoimmune Hepatitis

**DOI:** 10.14309/crj.0000000000002171

**Published:** 2026-06-11

**Authors:** Saman Namazian, Allison Bai, Erika Elliott, Mary Hopkins, Christopher Leung

**Affiliations:** 1 Tufts University School of Medicine, Boston, MA; 2 Department of Dermatology, Tufts Medical Center, Boston, MA; 3 University of Illinois College of Medicine, Chicago, IL; 4 Department of Infectious Diseases, Tufts Medical Center, Boston, MA; 5 Tufts Medicine Community Care Center, Stoneham, MA

**Keywords:** secondary syphilis, autoimmune hepatitis (AIH), syphilitic hepatitis, *Treponema pallidum*, liver biopsy, benzathine penicillin G

## Abstract

Syphilis is a systemic infection that can involve multiple organ systems, including the liver. Distinguishing between syphilitic hepatitis (SH) and autoimmune hepatitis (AIH) can be challenging, as both present with overlapping clinical, serologic, and histological features. We present a rare case of secondary syphilis acting as a potential trigger for AIH in a 42-year-old man. This case highlights how *Treponema pallidum* infection may unmask or exacerbate underlying immune dysregulation in susceptible individuals. SH was excluded based on clinical and pathologic correlation, including histology consistent with AIH and the absence of features supporting SH. Early recognition and appropriate treatment are pertinent, as the management of AIH differs from that of SH. The patient achieved sustained biochemical remission after corticosteroid therapy.

## INTRODUCTION

Syphilis is a multisystemic infection caused by the spirochete *Treponema pallidum*, with rising global incidence.^1^ Syphilitic hepatitis (SH), occurring in approximately 2.7% of secondary syphilis cases, results from hematogenous hepatic dissemination of *T. pallidum* and typically produces a cholestatic pattern of liver injury.^2,3^ Autoimmune hepatitis (AIH) is a chronic inflammatory liver disease characterized by immune-mediated hepatocyte destruction, with a prevalence of 16 to 18 per 100,000.^4,5^ Both conditions share overlapping serologic and histologic features, and infections including syphilis can trigger AIH in genetically susceptible individuals.^6^

The patient's baseline liver tests were normal 2 months before presentation, raising the possibility that *T. pallidum* infection contributed to autoimmune activation.

## CASE REPORT

A 42-year-old man with intravenous opioid use disorder (in remission on buprenorphine-naloxone) and previously treated hepatitis C (sustained virologic response) presented with progressive fatigue, weight loss, arthralgias, hair loss, blurred vision, angular cheilitis, and a diffuse rash on the trunk and upper extremities.

### Physical examination

**Figure 1.**
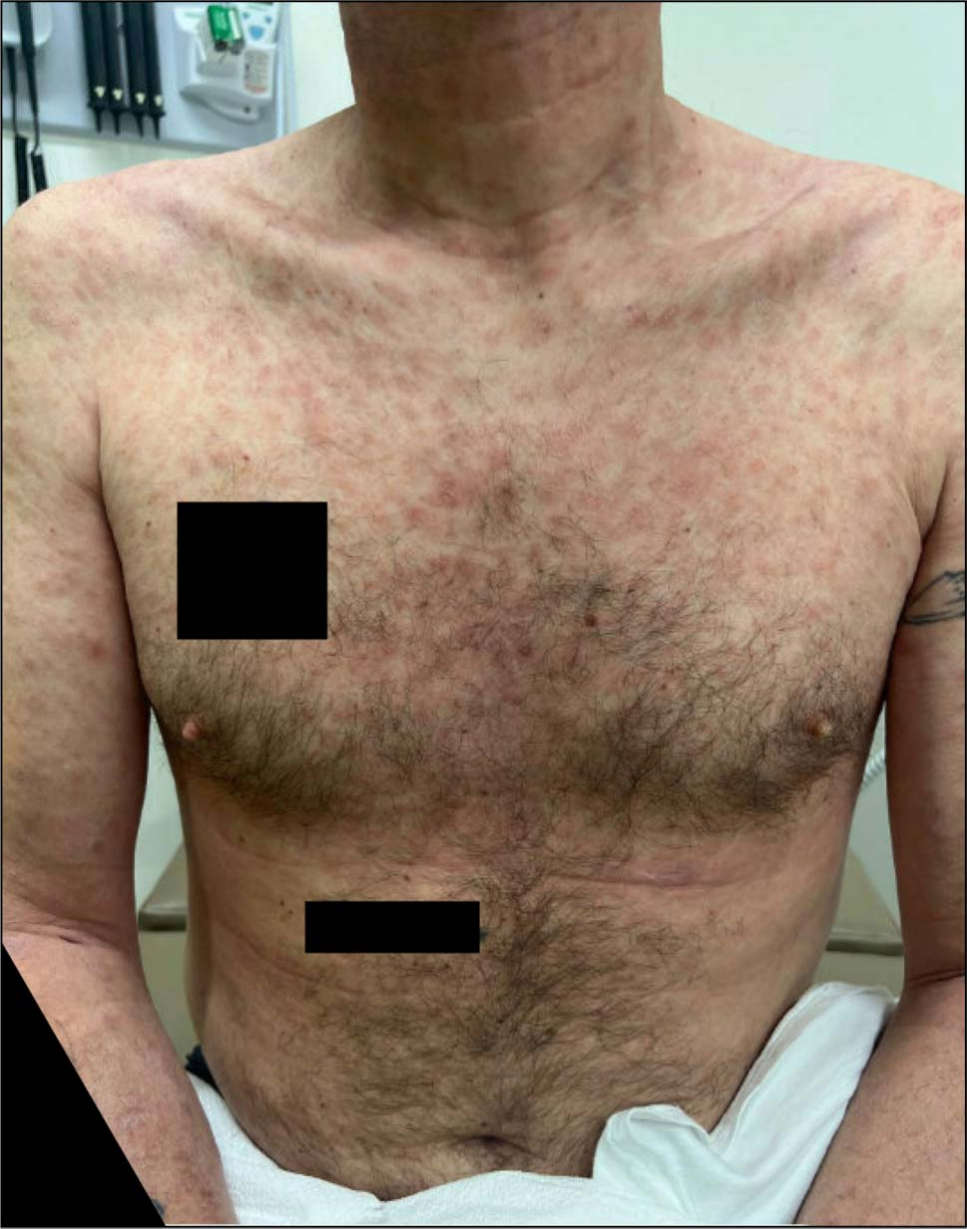
Scaly, erythematous, confluent plaques involving the scalp, trunk, and upper extremities on physical examination.

**Figure 2.**
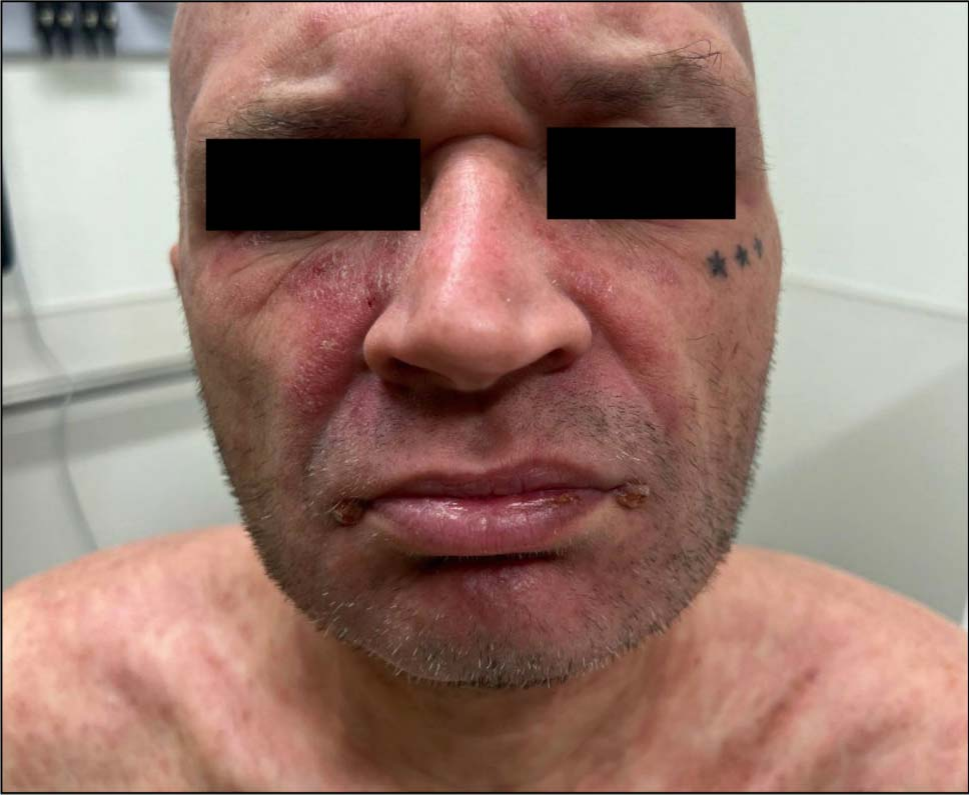
Angular cheilitis with split papules at the oral commissures seen on physical examination.

Examination revealed scaly, erythematous, confluent plaques on the scalp, trunk, and arms (Figure 1) and split papules at the oral commissures (Figure 2). No jaundice, hepatomegaly, or stigmata of chronic liver disease were noted.

### Medication and exposure history

Medications included buprenorphine-naloxone, cholecalciferol, fluconazole, omeprazole, phenylephrine, and sumatriptan. He denied alcohol, supplements, recent acetaminophen or nonsteroidal anti-inflammatory drug use, and had been abstinent from recreational drugs for over a year. No recent travel or occupational toxin exposure was reported.

### Laboratory findings

Laboratory findings at peak presentation included alanine aminotransferase 81 U/L (ref 7–56), aspartate transaminase 116 U/L (ref 10–40), alkaline phosphatase (ALP) 588 U/L (ref 44–147), and total bilirubin 1.0 mg/dL. Immunoglobulin G (IgG) was 3,250 mg/dL (ref 700–1,600) and IgM 465 mg/dL (mildly elevated, considered nonspecific). Antismooth muscle antibody was 1:320, antimitochondrial antibody (AMA)-M2 183.6 U (<20), and antinuclear antibody (ANA) negative. International normalized ratio was 1.1. Iron studies were normal; ceruloplasmin and alpha-1 antitrypsin were not obtained. Hepatitis C virus RNA was undetectable; hepatitis A, B, E, HIV, Epstein Barr virus, and cytomegalovirus were not tested due to low clinical suspicion. He had documented hepatitis B immunity. The overall pattern was mixed hepatocellular-cholestatic with disproportionate ALP elevation.

### Timeline of liver tests

Table 1 summarizes enzyme trends and key clinical events. Erythrocyte sedimentation rate was 78 mm/hr and C-reactive protein 8.53 mg/dL at peak.

**Table 1.** Timeline of liver enzymes and clinical events

Time point	ALP (U/L)	AST (U/L)	ALT (U/L)	Clinical event
2 months prior	Normal	Normal	Normal	Baseline laboratory results
Initial presentation	332	Normal	Normal	Syphilis testing sent
∼1 month later	∼700	∼100	∼100	Prednisone started (titrated to 40 mg daily)
∼1 week postprednisone	Declining	Declining	Declining	Benzathine penicillin G first dose
∼4 weeks postprednisone	Markedly decreased	Normalizing	Normalizing	Biochemical improvement confirmed
Several months later	Normal	Normal	Normal	Full remission achieved

ALP, alkaline phosphatase; ALT, alanine aminotransferase; AST, aspartate transaminase.

### Diagnosis and treatment

Given elevated IgG and high-titer autoantibodies, an AIH flare was diagnosed and prednisone started at 10 mg daily, titrated to 40 mg, before antibiotic initiation. Syphilis serologies returned an rapid plasma regain of 1:512 with reactive treponemal antibody, confirming secondary syphilis. Benzathine penicillin G 2.4 million units intramuscular was administered 3 times over 4 weeks, with the first dose given approximately 1 week after prednisone initiation.

### Liver biopsy

**Figure 3.**
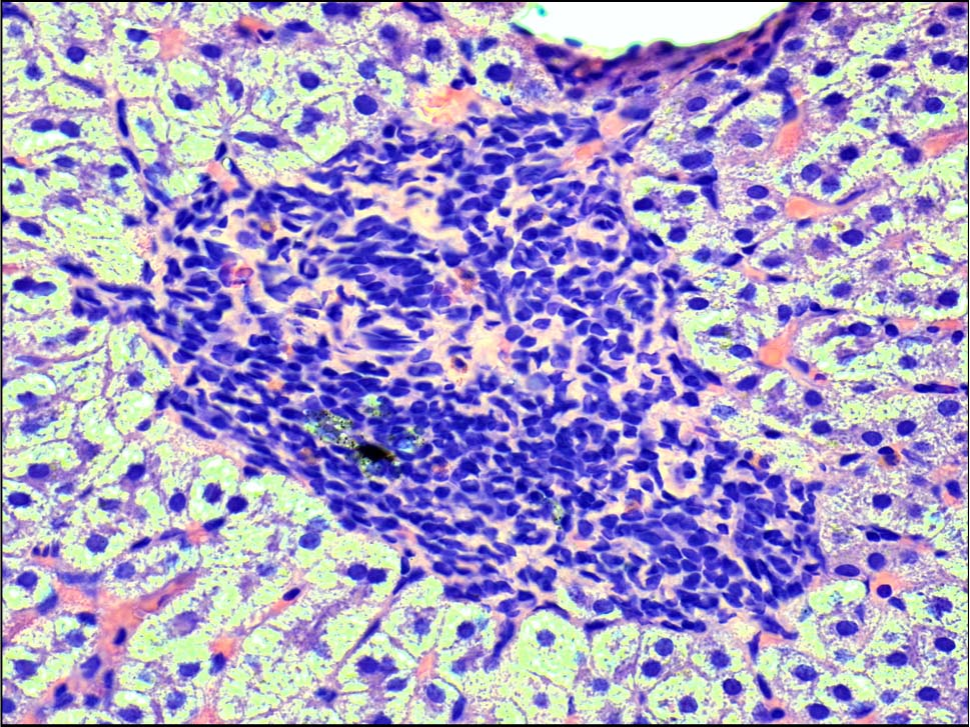
Liver biopsy demonstrating portal and periportal inflammatory infiltrates with plasma cell predominance on hematoxylin and eosin staining (original magnification ×40), consistent with autoimmune hepatitis.

Liver biopsy showed portal and periportal mononuclear inflammation with plasma cell predominance and scattered eosinophils (Figure 3). No steatosis, apoptotic necrosis, or bile duct injury was observed, consistent with AIH rather than syphilitic or cholestatic hepatitis.

### Clinical course

Aspartate transaminase, alanine aminotransferase, and ALP declined markedly within 4 weeks of corticosteroid initiation, with normalization of transaminases and IgG over subsequent months. At the 12-month follow-up, the patient remained in biochemical remission on a slow prednisone taper. Sustained remission was achieved without transition to azathioprine.

## DISCUSSION

Secondary syphilis presents with diffuse rash, mucosal lesions, and systemic symptoms.^1^ SH occurs in approximately 2.7% of cases and typically produces cholestatic liver enzyme elevation with marked ALP and gamma-glutamyl transferase rise.^7^ Histologically, SH may show portal infiltrates and spirochetes identifiable on silver or immunohistochemical staining.^8^

AIH presents with transaminase elevation, hypergammaglobulinemia, and characteristic autoantibodies (anti-smooth muscle antibody, ANA, or anti-liver-kidney microsomal antibody), with interface hepatitis on biopsy.^9–11^ Although ALP elevation and positive AMA can suggest AIH-primary biliary cholangitis overlap, biopsy showed no bile duct injury or ductopenia, arguing against it.^6^ Notably, while this patient met the Paris Criteria for AIH-primary biliary cholangitis overlap (sensitivity 92%, specificity 97%), the absence of florid duct lesions or destructive cholangitis on biopsy rendered true overlap unlikely.^12^ The elevated AMA-M2 and disproportionate ALP elevation are more plausibly attributed to the inflammatory milieu of concurrent syphilitic infection.

The biochemical profile was more consistent with AIH than SH. Biopsy showed no granulomas, and corticosteroid therapy produced a rapid decrease in enzymes before penicillin was initiated, inconsistent with isolated SH.^8,13^ The temporal overlap of treatments is a recognized limitation. However, sustained biochemical remission on corticosteroid monotherapy further supports an autoimmune rather than infectious etiology.^14^

*T. pallidum* may induce autoimmunity through molecular mimicry between spirochetal antigens and hepatic proteins, activating autoreactive lymphocytes.^15^ Infections can promote immune dysregulation and loss of tolerance, thereby unmasking latent autoimmune disease in genetically predisposed hosts.^16^

Our case adds to the limited literature on infection-induced AIH. Ali et al described biopsy-proven SH that resolved with antibiotics, only for the patient to return weeks later with hypergammaglobulinemia, positive autoantibodies, and interface hepatitis, suggesting that SH may trigger the autoimmune cascade.^14^ Riddell et al reported the first pediatric case of concurrent AIH and secondary syphilis, with elevated IgG, positive ANA, and biopsy-confirmed interface hepatitis.^17^ Unlike both reports, our patient lacked jaundice or fulminant enzyme elevation. Unlike Riddell et al, remission was achieved on corticosteroids alone without azathioprine. In contrast to both, prednisone preceded antibiotics and improvement preceded penicillin, more directly implicating autoimmunity as the dominant process.

Clinicians should consider AIH in patients with diffuse dermatitis, systemic symptoms, and persistent liver enzyme abnormalities, even with confirmed infection. Serologic testing, autoantibody profiling, and histologic evaluation are essential, as timely immunosuppression can prevent progression to chronic liver disease.

## DISCLOSURES

Author contributions: S. Namazian: Conception and design; literature review; drafting and critical revision of the manuscript; final approval. A. Bai: Conception and design; literature review; drafting of the manuscript; final approval. E. Elliott: Clinical care of the patient; acquisition of clinical data; critical revision for important intellectual content; final approval. M. Hopkins: Clinical care of the patient; acquisition of clinical data; critical revision for important intellectual content; final approval. C. Leung: Supervision; clinical care of the patient; critical revision for important intellectual content; final approval and is the article guarantor.

Acknowledgments: We thank Dr. Lei Duan from the Department of Pathology at Tufts Medical Center for reviewing the liver biopsy and providing the histology images used in this report.

Financial disclosure: None to report.

Informed consent was obtained for this case report.

## ABBREVIATIONS:

AIH: autoimmune hepatitis
ALP: alkaline phosphatase
ALT: alanine aminotransferase
AMA: anti-mitochondrial antibody
AST: aspartate aminotransferase
IgG: immunoglobulin G
SH: syphilitic hepatitis
